# Effect of Refining Degree on the Quality Changes and Lipid Oxidation of Camellia (*Camellia oleifera*) Oil during Heating

**DOI:** 10.3390/foods11152232

**Published:** 2022-07-27

**Authors:** Mei Wang, Yin Wan, Ting Liu, Xiuying Zeng, Xinmei Liang, Xiaojiang Wu, Guiming Fu

**Affiliations:** 1 State Key Laboratory of Food Science and Technology, College of Food Science and Technology, Nanchang University, Nanchang 330047, China; 352313318018@email.ncu.edu.cn (M.W.); yinwan@ncu.edu.cn (Y.W.); lxm1943487312@163.com (X.L.); 2 International Institute of Food Innovation, Nanchang University, Nanchang 330299, China; 3 State Center of Quality Testing and Inspection for Camellia Products, Ganzhou 341000, China; tliu0221@163.com (T.L.); zengxy3130@163.com (X.Z.); 4 Ganzhou General Inspection and Testing Institute, Ganzhou 341000, China; 5 College of Food Science, Shanxi Normal University, Taiyuan 030000, China; wuxiaojiang99@126.com

**Keywords:** camellia oil, lipid oxidation, refining degree, lipidomics

## Abstract

Refining degree has an important influence on the quality of camellia (*Camellia oleifera*) oil. The deterioration behaviors and lipid oxidation of three kinds of camellia oils, including camellia crude oil (CO), moderate refined oil (MRO), and refined oil (RO), during heating were investigated in this study. The results of deterioration behavior analysis showed that the oxidation degree was RO > CO > MRO. Tocopherol and polyphenolic substances in the oil might help delay oil oxidation. The lipid oxidation results indicated that the heating process had greater effects on CO and MRO than RO; it upregulated neutral lipid content and downregulated phospholipid content in terms of lipid changes and the multiplicity of differences. Glycerophospholipid metabolism was the most remarkable pathway and was important to study the heating process of refined oil. Moderate refining is good for retaining the beneficial lipids in camellia oil. The results of this study would provide a theoretical basis for camellia oil processing.

## 1. Introduction

*Camellia oleifera (C. oleifera)* is one of the four major woody oil plants in the world and is a primary source of edible vegetable oil [1,2]. *Camellia oleifera* is mainly distributed in the southern regions of China, such as Hunan, Jiangxi, Guangxi, Zhejiang, Anhui, and Fujian. Camellia oil, known as “oriental olive oil”, is extracted from *C. oleifera* seeds. Camellia oil has become a hot topic of research and received much interest owing to its richness in antioxidant active ingredients [3,4]. The improvement of people’s living standards and dietary structure has changed the demand for edible oils from quantity to quality; people have started to pursue edible oils with a high quality and high nutritional value and focused on the improvement of the production process.

Camellia crude oil contains abundant functional active ingredients, such as squalene, phytosterols, polyphenols, and fat-soluble vitamins, which have important roles in the oil’s nutritional and health effects [5]. However, crude oil easily becomes rancid, which makes the quality poor for consumption; therefore, the oil-processing industry refines crude oil. Oil refining is a series of processes that remove impurities from crude oil to improve the edibility and storage stability of the oil. Similar to other vegetable oils, the traditional refining method of camellia oil includes degumming, deacidification, decolorization, and deodorization [6]. On the one hand, refining can remove water and free fatty acids that affect the storage stability of the oil. On the other hand, it can also remove a large portion of the nutritional properties of the oil, which will reduce the oil quality. For example, refining could reduce the sterols, tocopherols, polyphenols, and essential fatty acids of camellia oil [7,8]. Chew and Ali attempted to control the refining conditions to retain the nutritional active ingredients, maintain the natural characteristics of edible oil, and meet food safety requirements [9]. For example, moderate refining, low-temperature refining, and a reduction in refining stages can better retain the antioxidant active substances and improve the oxidative and thermal stabilities of camellia oil. According to Wei et al. [7] and Chew et al. [10], low-temperature degumming can remove gums and preserve oil quality simultaneously. Lee et al. optimized the processing conditions during the degumming, alkaline refining, bleaching, and deodorization of crude camellia oil to obtain high-quality edible camellia oil [6].

Camellia oil has been considered “the king of cooking” because it contains over 85% unsaturated FA and high levels of active substances [11]. During heating, the oil undergoes many reactions, including hydrolysis, oxidation, and polymerization; these reactions degrade oil quality and produce free fatty acids, polar compounds, aldehydes, ketones, acids, and other substances that are hazardous to human health owing to the presence of oxygen and moisture [12]. The thermal oxidation stability of oil is an indicator of the resistance of an oil to some deterioration reactions that occur during high-temperature treatment [13]. Some natural antioxidant substances in camellia oil, such as tocopherol and polyphenols, can play a protective role in high-temperature treatment [14], but different refining processes can cause the loss of some of these antioxidants. Nonetheless, the effect of the refining process of camellia oil on its subsequent thermal cooking has been studied less widely. What happens to the different refined oils during the heat treatment process is not known. Therefore, in this study, physicochemical analysis and lipidomic techniques were used to study the oxidation degrees and lipid changes of three camellia oils with different refining degrees, namely, crude oil (CO), moderately refined oil (MRO), and refined oil (RO), during the heating process to explore the effects of the refining degree on camellia oil quality.

## 2. Materials and Methods

### 2.1. Materials

Camellia oil (acid value (AV)_CO_:1.10 mg/g, AV_MRO_:0.58 mg/g, AV_RO_:0.17 mg/g) was offered by Ganzhou Jinxi Agricultural Development Co., Ltd. (Ganzhou, China). Standard α-tocopherol, β-tocopherol, γ-tocopherol, δ-tocopherol, and fatty acid methyl ester were obtained from Tianjin Alta Technology Co., Ltd. (Tianjin, China). Chromatography-grade methanol, hexane, acetonitrile, dichloromethane, and methylene chloride were obtained from Merck Life Sciences (Shanghai, China) Co., Ltd. (Shanghai, China). All other chemicals used were of analytical grade.

### 2.2. Sample Preparation

Preparation of CO: The moisture of camellia seeds was controlled at about 8% after drying. Then, the seeds were cold-pressed with a two-screw press (SYZX 12, Hubei Anlu Tianxing Grain and Oil Machinery Equipment Co., Ltd., Anlu, China) to obtain CO.

Preparation of MRO: CO was filtered by a plate-type hermetic filter (NYB-4, Jiangsu Juneng Machinery Co., Ltd., Wuxi, China), the filtering temperature was 60~70 °C, the pressure was less than 0.3 MPa. Then, the oil temperature was raised to (50 ± 5) °C before the addition of 10% (*w*/*w*) of the oil amount of NaCl (3% *w*/*w*). The mixture was stirred for 30 min and left to precipitate for 2 h, the oil sediment was released, and the oil washed 1~2 times with water. The washed oil was injected into the bleaching pan (YSY100, Hubei Anlu Tianxing Grain and Oil Machinery Equipment Co., Ltd., Anlu, China), heated, and dried, before the addition of approximately 3% (*w*/*w*) of the oil amount of active white clay (Jiangxi Province Yushan County Lixin Bentonite Chemical General Factory, Shangrao, China), stirring for 30 min, and filtering after the oil temperature dropped to (70 ± 5) °C.

Preparation of RO: MRO was heated in a vacuum deodorization tower (YTST20, Hu-bei Anlu Tianxing Grain and Oil Machinery Equipment Co., Ltd., Anlu, China) to (250 ± 5) °C, injected with the appropriate amount of steam, kept warm for 2 h and then subjected to decreasing temperature to obtain refined oil. The refined oil was injected into the crystallizing kettle (YJY100, Hubei Anlu Tianxing Grain and Oil Machinery Equipment Co., Ltd., Anlu, China) and incubated at −5 °C for 48 h. After the oil formed crystals, it was filtered and separated.

CO (2.5 L) was poured into a magnetic stirring oil bath and heated at 170 ± 5 °C for 16 h at 8 h per day (4 h in the morning and 4 h in the afternoon). Oil samples (150 g) were collected every 2 h, placed in a brown sample bottle, cooled to room temperature, and reserved in a −20 °C refrigerator. MRO and RO were heated as mentioned. The CO, MRO, and RO obtained after heating for 16 h were named heated crude oil (HCO), heated moderately refined oil (HMRO), and heated refined oil (HRO), respectively. Untreated crude oil (UCO), untreated moderately refined oil (UMRO), and untreated refined oil (URO) were the corresponding control groups.

### 2.3. Basic Physical and Chemical Indicators

Color determination was performed according to GB/T 22460-2008. A fully automated Lovibond tintometer (TLV-100A, Hangzhou Daji Photoelectric Instrument Co., Ltd., Hangzhou, China) was used to determine the color values of the samples.

The iodine value was determined with reference to GB/T 5532-2008. A test portion was dissolved in solvent, added Wijs reagent. After a specified time, we added potassium iodide and water, and titrated the liberated iodine with sodium thiosulfate solution.

The fatty acid content was determined according to GB 5009.168-2016 (third method). The samples were saponified and methyl esterified in a 2% potassium hydroxide methanol solution to produce fatty acid methyl esters, which were analyzed by capillary column (SP-2560, 100 m × 0.25 mm, 0.2 μm, Supelco) gas chromatography (GC, TRACE 1300, Thermo Scientific, Milan, Italy) with a flame-ionization detector (FID) and quantified by the area normalization method to determine the percent fatty acid content. The operating conditions were: carrier gas high-purity nitrogen flow rate of 0.6 mL/min; initial temperature of 100 °C for 13 min; increase to 180 °C at a rate of 10 °C/min, hold for 6 min; increase at a rate of 1 °C/min 200 °C, hold for 20 min; increase to 230 °C at a rate of 4 °C/min, hold for 20.5 min; injection temperature 225 °C; flame ionization detector temperature 270 °C; detector temperature 280 °C.

The peroxide value (PV) was determined according to GB 5009.227-2016 (first method). The sample was dissolved in trichloromethane and glacial acetic acid, in which the peroxide reacted with potassium iodide to produce iodine, and the precipitated iodine was then titrated with a standard solution of sodium thiosulfate.

The *p*-anisidine values (*p*-AVs) were determined based on GB/T 24304-2009. A test solution was prepared in isooctane (2,2,4-trimethylpentane) and reacted with an acetic acid solution of *p*-anisidine. The increase in absorbance at 350 nm was measured. The anisidine value was calculated.

The K232 and K268 values were determined with reference to GB/T 22500-2008. A sample was dissolved in isooctane and the absorbance was measured spectrophotometrically at 232 nm and 268 nm.

The polar components were determined with an edible oil quality tester (Testo 270). We removed the oil from fryer and waited for about 5 min until no bubbles emerged from the oil bath to start measurements. We then immersed the probe vertically into the hot oil up to the center of the tank, with the immersion depth between the min/max mark. After gently stirring the probe, the results were read and recorded.

The tocopherol content was determined according to GB/T 26635-2011. A test portion was dissolved in n-heptane and the individual tocols were separated by an ultra-high pressure liquid chromatograph (UHPLC, Vanquish, Thermo Scientific, Germering, Germany) equipped with a diode array detector (DAD). A silica column (250 mm × 4.6 mm, 5 μm, Thermo, Sunnyvale, CA, USA) was used. The separation of tocopherols was performed at 292 nm using a volume fraction of 3.85% tetrahydrofuran n-heptane solution as the mobile phase with a flow rate of 1.0 mL/min. The sample injection volume was 10 μL. The column temperature was set at 20 °C.

The determination of polyphenols content was conducted according to LS/T 6119-2017. The polyphenols in the samples were purified by a diol-based column and quantified by the Folin–Ciocalteu (F–C) method using gallic acid as a calibration standard.

### 2.4. Lipid Extraction

The method of lipid extraction was followed according to Werner et al. with some modifications [15]. A 100 μL aliquot of each sample was transferred into a 2 mL centrifuge tube, mixed with 750 μL of chloroform–methanol mixed solution (2:1, pre-cooled at −20 °C), vortexed for 30 s, placed on ice for 40 min, mixed with 190 μL of H_2_O, vortexed for 30 s, placed on ice for 10 min, and centrifuged at 12000 rpm for 5 min at room temperature. A 300 μL aliquot of the lower-layer fluid was transferred into a new centrifuge tube, mixed with 500 μL of a chloroform–methanol mixed solution (2:1, pre-cooled at −20 °C), vortexed for 30 s, and centrifuged at 12000 rpm for 5 min at room temperature. A 400 μL aliquot of the lower-layer fluid was transferred into the same centrifuge tube. The samples were concentrated until dry in a vacuum. The dried samples were dissolved with 200 μL of isopropanol, and the supernatant was filtered through a 0.22 μm membrane to obtain the prepared samples for liquid chromatography–electrospray ionization–tandem mass spectrometry (LC–ESI–MS/MS). Quality control samples were used to monitor deviations in the analytical results from these pool mixtures, which were compared with the errors caused by the analytical instrument. The rest of the samples were used in LC–ESI–MS/MS detection.

### 2.5. LC–ESI–MS/MS Analysis

Chromatographic separation was performed with an Acquity UPLC^®^ BEH C18 column (100 mm × 2.1 mm, 1.7 μm, Waters, Milford, MA, USA) maintained at 50 °C. The autosampler temperature was 8 °C. Gradient elution was carried out with acetonitrile–water (60:40, 0.1% formic acid + 10 mM ammonium formate) (C) and isopropanol–acetonitrile (90:10, 0.1% formic acid +10 mM ammonium formate) (D) at a flow rate of 0.25 mL/min. A 2 μL aliquot of each sample was injected after equilibration. An increasing linear gradient of solvent C (*v*/*v*) was used as follows: 0–5 min, 70–57% C; 5–5.1 min, 57–50% C; 5.1–14 min, 50–30% C; 14–14.1 min, 30% C; 14.1–21 min, 30%–1% C; 21–24 min, 1% C; 24–24.1 min, 1%–70% C; 24.1–28 min, 70% C.

The ESI–MS experiments were performed with spray voltages of 3.5 and −2.5 kV in the positive and negative modes, respectively. Sheath gas and auxiliary gas were set at 30 and 10 arbitrary units, respectively. The capillary temperature was 325 °C. The Orbitrap analyzer was used to conduct a full scan over the mass range of m/z 150–2000 at a mass resolution of 35,000. Data-dependent acquisition MS/MS experiments were performed with a high-energy collisional dissociation scan. The normalized collision energy was 30 eV. Dynamic exclusion was implemented to remove unnecessary information in the MS/MS spectra [16,17].

### 2.6. Data Processing and Lipid Identification

The raw data (*.raw format) were annotated by LipidSearch software (version 4.2, Thermo Scientific, Waltham, MA, USA) to obtain a data matrix including the mass to charge ratio (*m*/*z*) and retention time (RT) and peak response value (intensity). The annotation results of all samples were aligned by LipidSearch software (version 4.2, Thermo Scientific, Waltham, MA, USA). Peak alignment and peak filtering were performed on the annotation results of all individual data, with the main parameter values as follows: RT tolerance = 0.25 and m-Score threshold = 3.

### 2.7. Data Analysis

Data normalization, principal component analysis (PCA), partial least squares discriminant analysis (PLS-DA), and orthogonal partial least squares discriminant analysis (OPLS-DA) were performed with the R package, MetaboAnalystR (3.0, Xia Lab, Montreal, Canada) [18]. The Normalization function in the MetaboAnalystR package (with arguments MedianNorm, LogNorm, and AutoNorm) was adopted to make the data close to a normal distribution. Univariate analyses (t-tests) were applied to calculate the statistical significance (*p*-value). The lipids with a value importance in projection (VIP) > 1, *p* < 0.05, and a log_2_ (fold change [FC]) > 1 were considered differential lipids. For clustering heat maps, the data were normalized as z-scores and plotted by the Pheatmap package in R. A volcano plot was used to filter the lipids of interest based on log_2_(FC) and −log10 (*p*-value), through the ggplot2 package in R. The lipids with *p* < 0.05 (t-test) were used in the over-representation analysis (ORA) enrichment analysis, and the resulting KEGG pathways with *p* < 0.05 (ORA) were considered to have a statistically significant enrichment.

### 2.8. Statistical Analysis

All analytical measurements were performed in triplicate, and the results were expressed as the mean ± standard deviation (SD). The data were fitted and plotted using Origin 9.0 (OriginLab, Northampton, MA, USA). Comparisons among groups were performed by one-way ANOVA followed by the least significant difference test (IBM SPSS 22.0, SPSS Inc., Chicago, IL, USA). *p* < 0.05 was considered statistically significant. The Pearson correlation was analyzed with SPSS 22.0.

## 3. Results and Discussion

### 3.1. Physical Parameters and Fatty Acid Composition

#### 3.1.1. Color Variation

**Figure 1:**
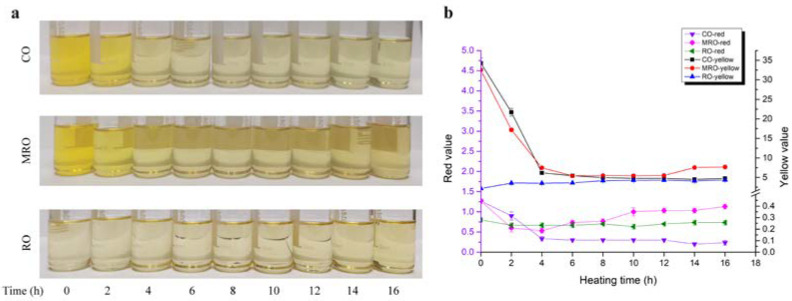
Changes in color in three kinds of camellia oil during heating. CO: crude oil; MRO: moderately refined oil; RO: refined oil. (**a**) Pictures of camellia oil samples during heating. (**b**) Changes in the red and yellow values of camellia oil samples during heating.

The color variations of the camellia oils during heating are shown in Figure 1a. CO and MRO had a lighter color during heating, probably because the fat-soluble pigments, such as chlorophyll and carotene, dissolved in the oil were easily decomposed when they encountered a high temperature. β-carotene is destroyed into colorless, inactive products [19], whereas chlorophyll is converted to demagnetized chlorophyll [20] at high temperatures. There was a significant increase in red values around 4 h and 10 h for MRO compared to CO and RO, which may be the result of oxidation reactions with oxygen dissolved by the high water content in MRO. Many studies have found that the color change in oils after heating tests is closely related to their phospholipid [21] and pigment contents [22]. RO underwent a decolorization stage during the refining process, and many pigments were adsorbed by substances, such as white clay; therefore, the color change during the heating process was not remarkable and was relatively stable. The trend presented in the Lovibond colorimetry determination results (Figure 1b) was consistent with the color change.

#### 3.1.2. Iodine Value

The iodine value is used to evaluate the unsaturation degree of oil. A higher iodine value indicates a greater degree of unsaturation. The iodine value of oil will change when heated because of the oxidation reaction or polymerization reaction of unsaturated fatty acids (UFAs). As a result, the unsaturated degree of fatty acids in the oil is reduced in the process of heating. However, the change in iodine value can only explain the change in the total UFAs in the oil and cannot specifically reflect the change in UFA content [23].

**Figure 2:**
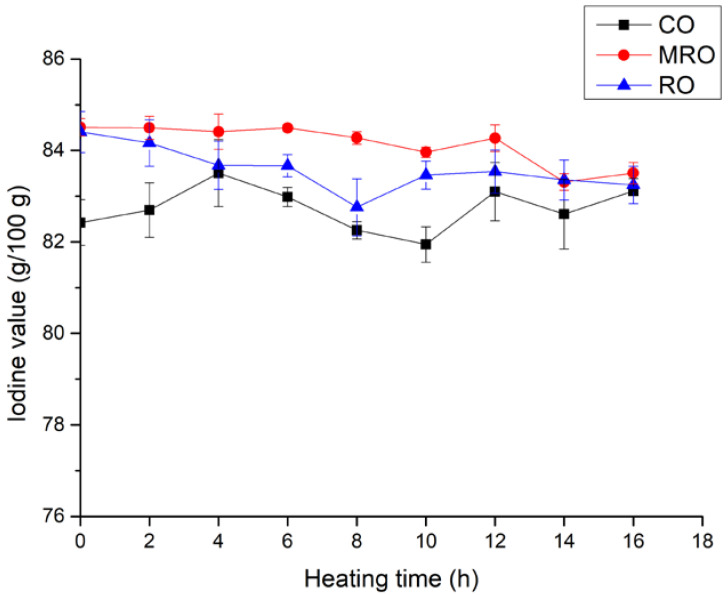
Changes in iodine value of three kinds of camellia oil during heating.

Changes in the iodine values of the three kinds of camellia oil during heating are shown in Figure 2. The initial iodine value of RO was the highest, whereas that of CO was the lowest, indicating that the UFA content of RO was higher. The iodine values of the three kinds of camellia oil did not change substantially during the heating process. The iodine values of MRO and RO decreased by 1.00 and 1.16 g per 100 g, respectively. The iodine value of CO showed a fluctuating increasing trend from 82.42 g/100 g to 83.11 g/100 g which might be due to the relatively stable composition of fatty acids. The iodine values of MRO exceeded the other two oils for most of the heating time, indicating that the MRO had a higher UFA content.

#### 3.1.3. Fatty Acid Composition

**Figure 3:**
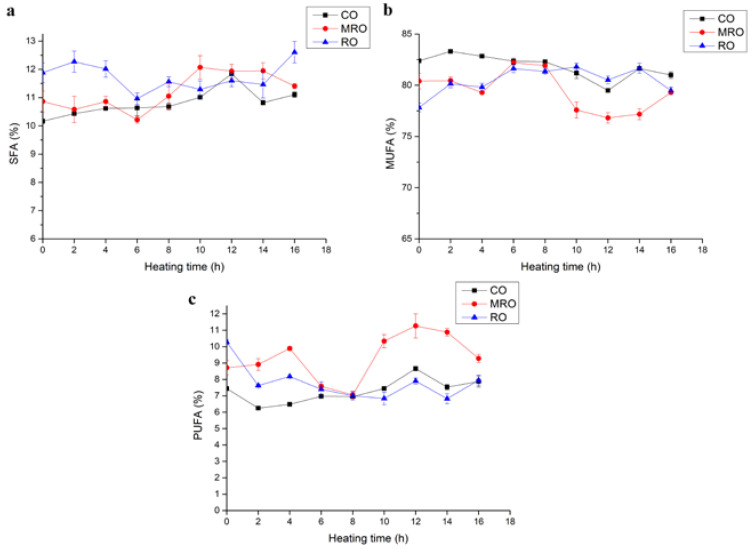
Changes in fatty acids in three kinds of camellia oil during heating. (**a**) Saturated fatty acids (SFAs); (**b**) monounsaturated fatty acids (MUFAs); (**c**) polyunsaturated fatty acids (PUFAs). Note: SFA is equal to the sum of myristic acid, palmitic acid, stearic acid, and arachidonic acid. MUFA is the sum of palmitoleic acid, oleic acid, arachidonic acid, erucic acid, and nervonic acid. PUFA is equal to the sum of linoleic acid and linolenic acid.

The initial fatty acid compositions of three kinds of camellia oil are shown in Table 1. For the sake of brevity, we have summarized the fatty acids into saturated, monounsaturated, and polyunsaturated fatty acids. The changes in the fatty acid compositions of the three kinds of camellia oil during heating are shown in Figure 3. The contents of the saturated fatty acids (SFAs), monounsaturated fatty acids (MUFAs), and polyunsaturated fatty acids (PUFAs) of the three kinds of camellia oil showed no obvious changes during heating but fluctuated up and down within a small range, which was similar to the change trend of iodine values during heating. This result indicated that the oxidation and hydrolysis reactions of oils during 16 h heating were not violent [24]. MRO had a slightly higher PUFA content than the other two oils during heating. This phenomenon echoed the change in iodine values, indicating that MRO had a better retention of UFAs during heating.

### 3.2. Degree of Oxidation

#### 3.2.1. PV, *p*-AV, and Total Oxidation (TOTOX) Value

**Figure 4:**
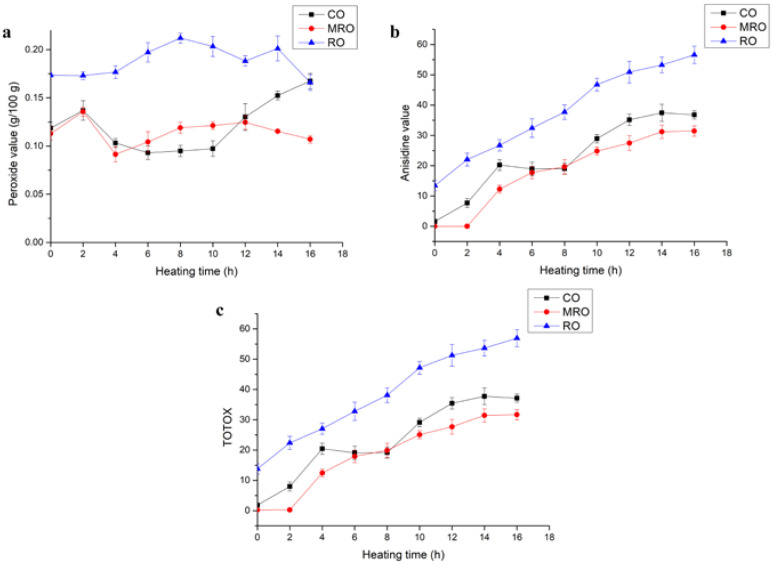
Changes in the (**a**) PV, (**b**) *p*-AV, and (**c**) TOTOX values of three kinds of camellia oil during heating. TOTOX: total oxidation values.

Peroxides (PVs) are an indicator of the content of primary oxidation products in fats and oils. During the heating process, complex oxidation reactions occur, resulting in unstable primary oxidation products (peroxides and hydroperoxides), which cause the peroxide value of oils to rise. The primary oxidation products will further react under high-temperature conditions to produce ketones, aldehydes, and other harmful substances, resulting in oil deterioration. *p*-AV is a measure of the amount of aldehydes (mainly alpha- and beta-unsaturated aldehydes) in oil. The higher the *p*-AV, the more severe the deterioration. *p*-AV reflects the change in secondary oxidation products. We introduced TOTOX (TOTOX = 2PV + *p*-AV) to better evaluate the overall oxidation level of oil. The TOTOX values are closely related to the freshness of oils; the lower the TOTOX value, the higher the freshness of the oil [25,26,27,28]. Changes in the PV (a), *p*-AV (b), and TOTOX values (c) of the three kinds of camellia oil during heating are shown in Figure 4.

Oil oxidation is a complex process. Primary oxidation products are formed when oil is heated. The primary oxidation products are unstable and continue to oxidize at high temperatures to produce substances containing carbonyl groups and aldehydes, leading to fluctuations in PV. In this experiment, the PV did not fluctuate considerably. The PVs of all three kinds of camellia oil fluctuated between 0.05 and 0.25 g/100 g. In particular, the PV of MRO was relatively low in the heating process, fluctuating around 0.1 g/100 g. The results show that the primary oxidation products of the three kinds of oil are in constant change during heating.

The *p*-AVs of CO and MRO were considerably lower than that of RO during the heating process, which might be related to the antioxidant active substances contained in CO and MRO. The initial *p*-AV in RO was higher than 10, which might be related to the high temperature of the deodorization process over a long time. The *p*-AV of all three oils increased slowly as the heating time increased. RO still had the highest *p*-AV and MRO had the lowest *p*-AV after 16 h of heating.

The TOTOX values of all three kinds of camellia oil showed an increasing trend during the heating process as presented in Figure 4c. The TOTOX values of RO were higher than those of CO and MRO, and the TOTOX values of MRO were the lowest, indicating that the oxidative degree of the three kinds of oil was: MRO > CO > RO. The rate of increase in the TOTOX values of RO was also faster than those of CO and MRO, probably because RO was fully refined, and substances with antioxidant functions, such as polyphenols and tocopherol, were removed from RO. As shown in the indicators of primary oxidation products, secondary oxidation products, and the overall oxidation level, MRO had the lowest oxidation degree during heating, followed by CO, and RO had the highest oxidation degree. This result may be related to the fact that MRO retained more natural antioxidant substances and removed a part of its impurities.

#### 3.2.2. K232 and K268

**Figure 5:**
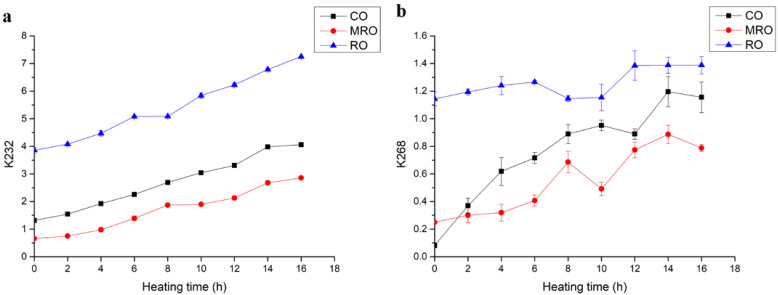
Changes in (**a**) K232 and (**b**) K268 in three kinds of camellia oil during heating.

During the heating of oil, the primary oxidation products produced are unstable and further degraded into secondary oxidation products: conjugated dienes and conjugated trienes. Conjugated alkenes are produced by the displacement of allyl on the PUFA chain in the triglyceride molecule during oxidation [29]. The variation in conjugated dienes with heating time is shown in Figure 5a. In the starting phase, MRO had the lowest K232 content, followed by CO, and RO had the highest K232 content, which is probably related to its refining process. As the heating time increased, the degree of oxidation reaction increased, and the value of conjugated dienes became higher. The conjugated alkenes of all three kinds of camellia oil accumulated gradually and showed an increasing trend. MRO still had the lowest K232 value, indicating the least oxidation, followed by CO, and MRO did not exceed the starting K232 value for RO after 16 h of heating. After 16 h of heating, RO had the highest K232 value, indicating that it had the greatest degree of oxidation.

The variation in conjugated trienes with heating time is shown in Figure 5b. CO had the lowest K268 in the starting phase, followed by MRO, and RO had the highest K268. As the heating time increased, MRO had the lowest K268, and the K268 values of the three kinds of oil initially increased, then decreased, and increased again. Conjugated trienes may be produced by the dehydration of conjugated dienes [30]. The secondary oxidation products produced at a later stage were further degraded or formed other small-molecule volatiles. These reasons may have contributed to the fluctuating rise of the conjugated trienes during heating.

The analyses of K232 and K268 values showed that the oxidative degree of the three kinds of camellia oil was in the order: RO > CO > MRO. MRO retained more antioxidant active substances and removed more impurities; hence, it had the best oxidative stability. CO had more antioxidant active substances but contained more impurities, which make oxidants accumulate partially. The oxidation stability of RO is weak because of its high degree of refining and low antioxidant activity, which is consistent with previous TOTOX results.

#### 3.2.3. Polar Components

Polar compounds are the thermal oxidation products of triglycerides, are more polar than normal triglycerides in oils, and are the main components of non-volatile products formed in oils during heating and frying [31]. The content of polar compounds can reflect the deterioration degree of oil during heating [32].

**Figure 6:**
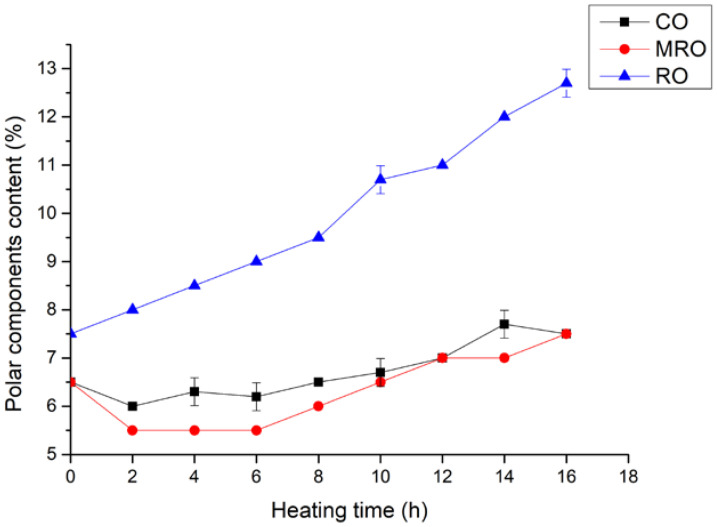
Changes in polar components content in three kinds of camellia oil during heating.

The changes in the contents of polar compounds in the three kinds of camellia oil during heating are shown in Figure 6. The content of polar compounds in all three kinds of camellia oil gradually increased with the extension of heating time. This finding was consistent with the research results of Houhoula et al. [33] and Song et al. [34]. The polar components in RO increased most obviously from 7.5% to 12.7%, and the content of polar compounds increased substantially after 8 h. The contents of polar compounds in CO and MRO showed little difference and increased slowly. Similar to previous results, the increase in the contents of polar compounds indicated that the quality of the three kinds of camellia oil decreased to different degrees during heating, but that of RO decreased the fastest.

### 3.3. Natural Antioxidant Substances

#### 3.3.1. Tocopherol

**Figure 7:**
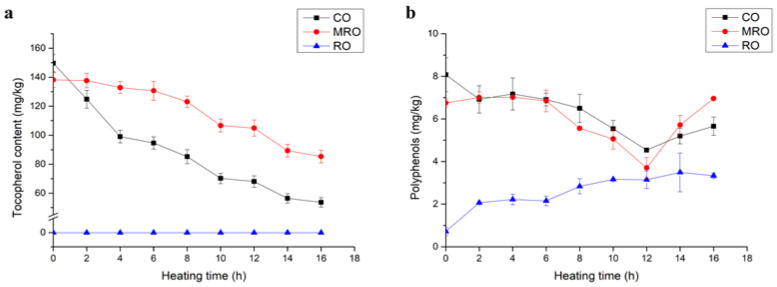
Changes in (**a**) tocopherol and (**b**) polyphenols contents in three kinds of camellia oil during heating.

The variations in the tocopherol contents of the three kinds of camellia oil during heating are shown in Figure 7a. In the initial stage, CO had the highest tocopherol content, followed by MRO, and the tocopherol content of RO was lower than the detection limit. The tocopherol content was related to the degree of refining of these three oils. Tocopherol content gradually decreased with the increase in heating time. Tocopherol decreased in CO rapidly. Tocopherol is unstable and is easily destroyed under high temperatures, oxygen, light, and other conditions. It is lost in high-temperature refining, cooking, and frying, and even affects the color and oxidation stability of oil. This result corroborated the previous oxidation results. MRO had the highest tocopherol content and the lowest oxidation degree owing to its antioxidant effect, whereas RO had almost no tocopherol and had the highest oxidation degree, and CO was intermediate between the two.

#### 3.3.2. Polyphenols

The presence of phenolic compounds in vegetable oil can effectively inhibit the formation of toxic thermal oxidation degradation compounds, help prolong the life of oil, and maintain the quality of oil [35,36]. Figure 7b shows that the polyphenol content of CO without heating was the highest, followed by MRO, and that of RO was the lowest.

This result was related to the refining degree of the three kinds of oil. During the heating process, the polyphenol content of CO decreased slightly with fluctuation, the polyphenol content of MRO exhibited up and down fluctuations but remained the same, and the polyphenol content of RO showed an increasing trend with fluctuations. The change in the content of free phenols is a dynamic process, which may be due to the fact that the heating process contributes to the leaching of free phenolic compounds, and the structure of bound phenols in oils and fats is destroyed and decomposed into free phenols at high temperatures, leading to an increase in the content of free phenols in oils [37]. After 16 h of heating, the polyphenol content was still the highest in CO, followed by MRO, and RO.

Tocopherol is a natural antioxidant and has an important influence on oxidative stability; it can protect polyunsaturated fatty acids, delay the oxidative rancidity of vegetable oil, and extend shelf life [38]. Polyphenolic substances might help delay oil oxidation. Many scholars have found that phenols and tocopherols play a good stabilizing role in the study of frying processes in oils [14,39]. Table 2 shows the remarkable positive correlation between tocopherols and polyphenols. The changes in tocopherols and polyphenols during heating were remarkably and negatively correlated with the changes in TOTOX values, conjugated olefins, and polar components. This result suggested that, similar to previous studies, tocopherols and polyphenols might help delay oil oxidation.

### 3.4. Lipidomic Analysis of Lipid Changes in Camellia Oil after Heating

#### 3.4.1. Identified Lipids Based on LC–ESI–MS/MS

A large number of oxidized lipids were produced by the three kinds of camellia oil owing to the complexity and diversity of lipids in camellia oil, especially after heating. Therefore, we adopted an efficient non-targeted lipid analysis method based on LC–ESI–MS/MS. Full-scan MS was used to detect as many lipids as possible using the ESI+ and ESI− modes, and MS/MS was used to obtain the secondary MS information of the lipids.

**Figure 8:**
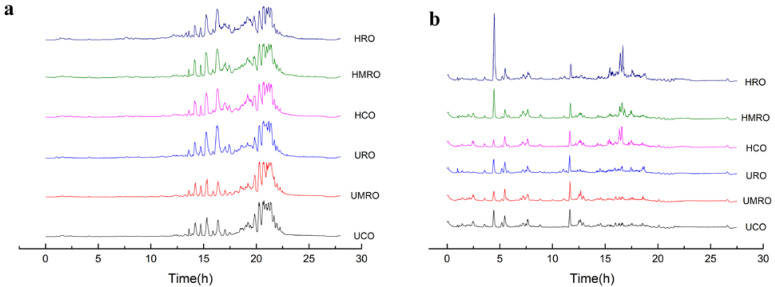
Total ion chromatography (TIC) of camellia oil before and after heating based on LC–ESI–MS/MS. (**a**) TIC in ESI+ mode; (**b**) TIC in ESI− mode). The CO, MRO, and RO obtained after heating for 16 h were named heated crude oil (HCO), heated moderately refined oil (HMRO), and heated refined oil (HRO), respectively. UCO: untreated crude oil. UMRO: untreated moderately refined oil. URO: untreated refined oil.

The results of the total ion chromatography of camellia oil before and after heating based on LC–ESI–MS/MS are shown in Figure 8. Elution in reversed-phase LC is based on the size of equivalent carbon number (equivalent carbon number = total carbon number of aliphatic acyl-2 double bond number); the larger the equivalent carbon number, the longer the retention time [40].

**Figure 9:**
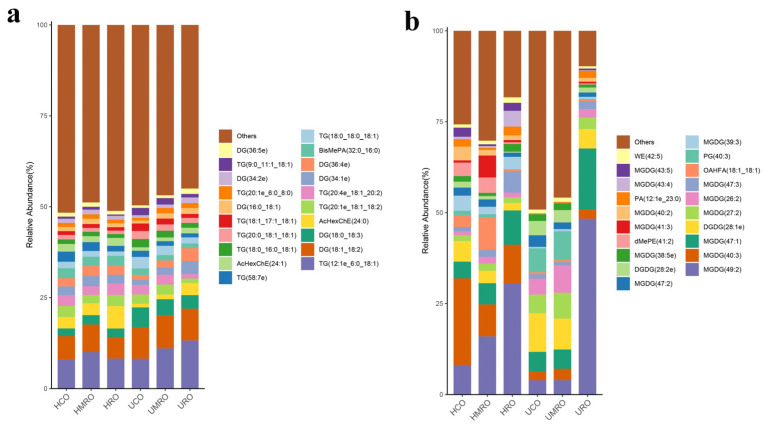
Barplot of camellia oil before and after heating based on non-targeted lipidomics: (**a**) positive ion mode; (**b**) negative ion mode.

A total of 1598 lipids were identified from the six kinds of camellia oil. Before heating, 1492 lipids of 42 kinds, 1518 lipids of 43 kinds, and 1499 lipids of 39 kinds were identified in UCO, UMRO, and URO, respectively. After heating, 1528 lipids of 41 kinds, 1547 lipids of 41 kinds, and 1483 lipids of 40 kinds were identified in HCO, HMRO, and HRO, respectively. Triacylglycerol (TG) and diacylglycerols (DG) were the main lipid types in the positive ion mode, whereas monogalactosyl diacylglycerols (MGDG) was the main lipid in the negative ion mode. The changes in lipid content in the negative ion mode were greater than those in positive ion mode. The lipid percentages of the six kinds of camellia oil with different degrees of refining before and after heating are presented in the visual accumulation histogram in Figure 9. The figure shows the top 20 lipids in terms of content, and the rest are included in “others”, which can intuitively compare the differences in lipid composition and structure between groups.

A total of 51 and 39 new lipids were produced during the heating of HCO and HMRO, respectively. Both oils had similar new lipids (TGs and DGs), with TG accounting for more than 50% and DG accounting for about 10%. Few new lipids were produced in HRO; these included DG, OAHFA, and phosphatidic acids (PA). We searched the lipid maps (https://www.lipidmaps.org/, accessed on 14 April 2022), KEGG (https://www.kegg.jp/, accessed on 14 April 2022), and MetaboAnalyst (https://www.metaboanalyst.ca/, accessed on 14 April 2022) databases to infer the possible lipid changes in camellia oil during heating. TG may be derived from other species of TG changes during heating or possibly from the reaction between diacylglycerol and carboxylate. DG may be derived from other DG changes or from TG hydrolysis. The additional MGDG may be derived from 1,2-DG via glycerolipid metabolism, and PA may be derived from 1,2-DG or Acyl-CoA via glycerolipid metabolism or from PC or PE via glycerophospholipid metabolism.

#### 3.4.2. Lipids Changes in Camellia Oils after Heating

The three kinds of oil lost different types of lipids. The lipids in HCO and HMRO that disappeared were glycerophospholipids, and the lipids in HRO that disappeared were glycerolipids and glycerophospholipids. HCO lost ceramides (Cer), monolysocardiolipin (MLCL), phosphatidylserines (PS), and lyso-phosphatidylethanolamines (LPE); HMRO lost Cer, phosphatidylethanolamines (PE), PS, phosphatidylglycerols (PG), and LPE; and HRO lost TG, DG, PE, and Cer. Cer may be involved in sphingolipid metabolism and act as an intermediate to produce sphingosine or ceramide 1-phosphate. PE may be involved in glycerophospholipid metabolism to produce PC, PS, LPE, and fatty acids. MLCL may be involved in glycerophospholipid metabolism as a precursor to produce cardiolipin. LPE may be involved in glycerophospholipid metabolism as a precursor to produce PE and may be interconverted with PE. PS may be involved in glycerophospholipid metabolism as a precursor to produce PE. LPE may be involved in glycerophospholipid metabolism as a precursor to PE and may be interconverted with PE. PS may produce PE, LPS, and fatty acids during heating. DG and fatty acids may be produced from the disappearance of TG. DG may be a precursor to PA, PE, PC, TG, MG, Cer, and fatty acids. The lipids that appeared and disappeared are detailed in Table 3 and Table 4.

#### 3.4.3. Clustering Analysis

**Figure 10:**
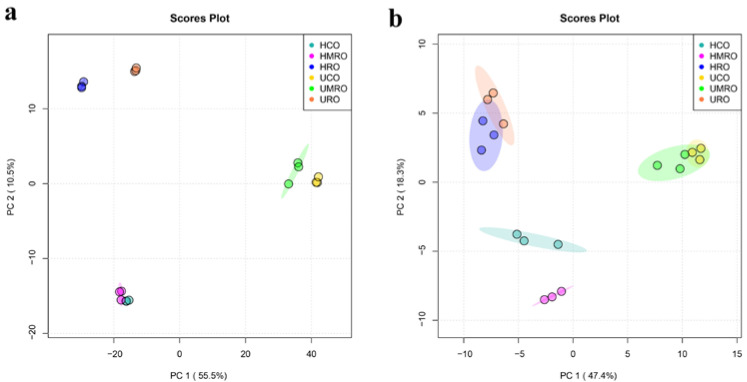
PCA scores plots of camellia oil before and after heating based on non-targeted lipidomics: (**a**) positive ion mode; (**b**) negative ion mode.

PCA is a multivariate statistical analysis method for unsupervised learning. PCA projects high-dimensional data into lower-dimensional space and retains information from the original data as much as possible [41], which can be used for the preliminary analysis of samples. The PCA results of the untreated and heated camellia oils are shown in Figure 10. The results showed that the cumulative contribution rates of principal components (PCs) one and two in positive ion mode were 66%, and those of PCs one and two in negative ion mode were 65.7%. Both values were greater than 60%, indicating that the model had a high coverage degree and good explanatory ability. The PCA score chart shows that six kinds of camellia oil can be clearly distinguished before and after heating in positive and negative ion modes. Taking the negative ion model as an example, the figure shows that UCO and UMRO were clustered together before heating, and HCO and HMRO were close together after heating but slightly further apart than before. This result indicated that the compositions of UCO and UMRO were relatively similar before heating but slightly changed after heating. URO and HRO clustered together before and after heating, indicating little change in the composition of RO before and after heating. However, RO was far away from CO and MRO, indicating that the composition of RO was quite different from those of CO and MRO. Therefore, the PCA results indicated that refining and heating can produce certain changes in the lipids of camellia oil.

**Figure 11:**
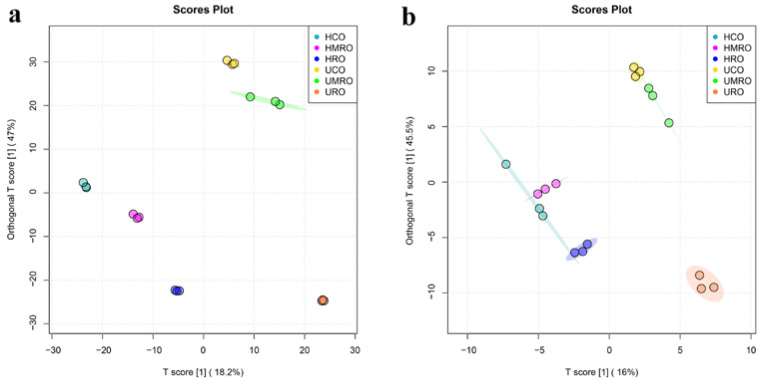
OPLS-DA scores plots of camellia oil before and after heating based on non-targeted lipidomics: (**a**) positive ion mode; (**b**) negative ion mode.

PLS-DA uses all components of the lipid data for prediction, which often results in more severe overfitting. In OPLS-DA, the regression model is built between the grouping information and the lipidomics data in the grouping information, and the model filters out information that is not relevant to the grouping [42]. Figure 11 shows that the three kinds of camellia oil were better separated under the positive ion mode (Figure 11a) and negative ion mode (Figure 11b) for untreated and heat-treated samples. In OPLS-DA, R^2^Y (cum) and Q^2^ (cum) are used to measure the discrimination effect of the model, and R^2^X (cum) is not commonly used. R^2^Y (cum) and Q^2^ (cum) denote the explanatory and predictive ability of the model, respectively [43]; an R^2^Y (cum) and Q^2^ (cum) greater than 0.5 indicate that the explanatory and predictive powers of the model are good, respectively [44]. The closer the R^2^Y (cum) and Q^2^ (cum) are to 1, the better the model differentiation is. The analysis results showed that the R^2^Y (cum) of the OPLS-DA model in the positive ion mode was 0.993, and Q^2^ (cum) was 0.946; the R^2^Y (cum) of the OPLS-DA model in the negative ion mode was 0.961, and Q^2^ (cum) was 0.851, which indicates that the explanatory and predictive abilities of the model were good in both the positive and negative ion modes.

**Figure 12:**
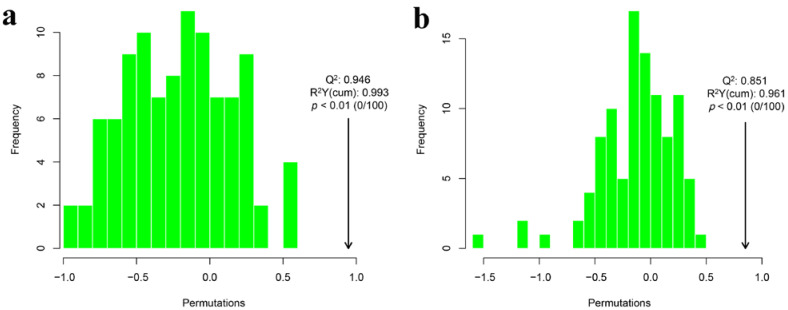
Non-targeted lipidomics test statistics: (Q^2^) distribution, the R^2^Y (cum), and *p*-value of OPLS-DA replacement test for camellia oil before and after heating. (**a**) Positive ion mode; (**b**) negative ion mode.

Sometimes, using the OPLS-DA score plot to model the discriminant effect is not convincing. At this time, permutation tests can be applied to determine whether the discriminant effect of our OPLS-DA discriminant model is due to completely random factors. Figure 12 displays the result of the permutation test of OPLS-DA using Q^2^ as the test statistic. The random distribution of Q^2^ was obtained by permutation. The actual observed Q^2^ values indicated by the arrows in the positive and negative ion modes were on the right side of the random distribution, and the observed values were significantly larger than the random value with *p* < 0.01. This result indicated that Q^2^ was not random and was significant, and the predictive power of the model was significant, which means that the lipids had significant differences between groups.

#### 3.4.4. Screening of Differential Lipids

**Figure 13:**
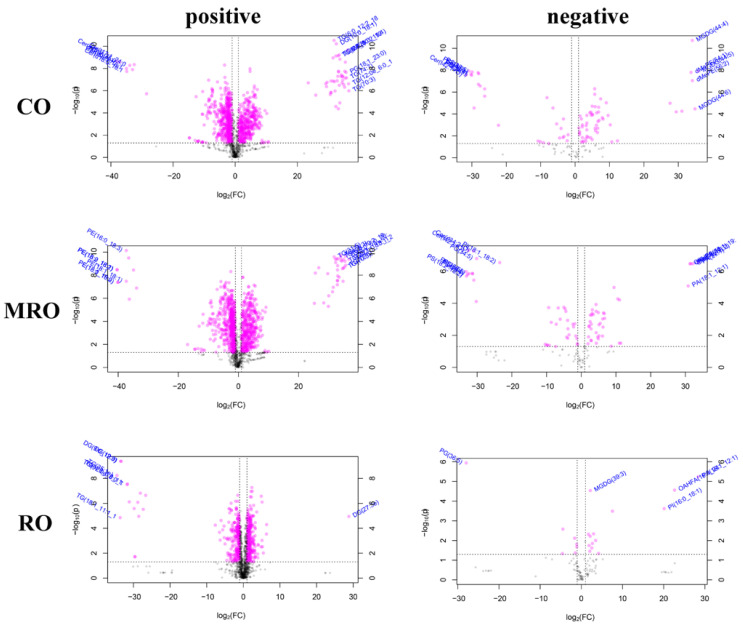
Volcanic diagrams of camellia oil before and after heating based on non-targeted lipidomics.

The magnitude of lipid oxide change was measured by calculating the FC of lipids, usually expressed as log_2_(FC), with an upward adjustment being positive and a downward adjustment being negative to further assess the lipid oxide change. Figure 13 shows the volcanic diagrams of the differential lipids in the three camellia oils with significant differences before and after heating. A total of 83 differential lipids in CO before and after heating were screened using VIP > 1.155, *p* < 0.05, and FC > 2.0 or FC < 0.5 as the thresholds, including 37 kinds under the positive ion mode and 46 kinds under the negative ion mode. A VIP > 1.1318, *p* < 0.05, and FC > 2.0 or FC < 0.5 were used as the thresholds to screen differential lipids in MRO before and after heating, and 90 species were screened, including 36 kinds in the positive ion mode and 54 kinds in the negative ion mode. With VIP > 1.275, *p* < 0.05, and FC > 2.0 or FC < 0.5 as the thresholds, 94 differential lipid oxides were screened in RO before and after heating, including 78 lipids under the positive ion mode and 16 lipids under the negative ion mode. The results are shown in Table 5. According to the table, neutral lipids were the main lipids in the three kinds of camellia oil before and after heating, followed by glycoglycerolipids, phospholipids, fatty acyl, and others.

##### Common Differential Lipids

**Figure 14:**
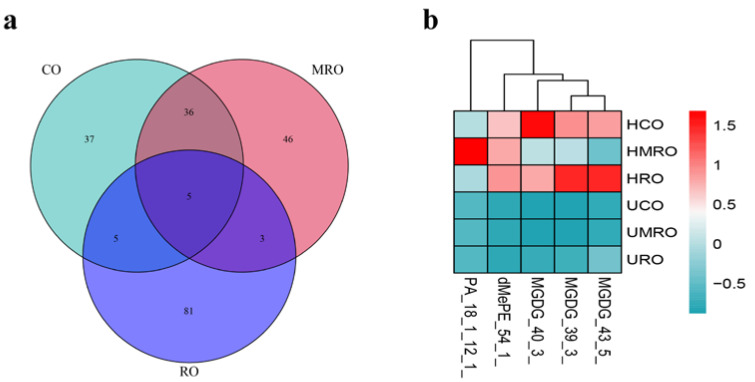
The Venn diagram and heat map of three kinds of camellia oil. (**a**) The Venn diagram; (**b**) The heat map.

The lipid changes of the same oil before and after heating were compared to filter out the differential lipids, and then the differential lipids of the three kinds of oil were compared to derive the common differential lipids. As shown in the Venn diagram in Figure 14a, five differential lipids were common to all three oils. The heat map (Figure 14b) shows that four of the five common differential lipids (MGDG [43:5], MGDG [39:3], MGDG [40:3], and dimethylphosphatidylethanolamine [dMePE, 54:1]) varied more in HCO and HRO than in HMRO, whereas one differential lipid (PA [18:1_12:1]) varied the most in HMRO.

##### Unique Differential Lipids

Figure 14 demonstrates that unique differential lipids were the most abundant in RO (81 lipids), followed by MRO (46 lipids), and CO (37 lipids). The distribution of unique differential lipids in the three kinds of camellia oil is shown in Table 6. Table 7 lists the differential lipids specific to CO, which include 14 TGs, 6 MGDGs, 4 MLCLs, and 3 OAHFAs. In total, 12 of the 37 differentials lipid were downregulated, and 25 were upregulated. The differential lipids with the highest degrees of upregulation and downregulation in CO were wax esters (WE, 8:0_19:4, 36.19) and Cer (d18:0_18:1, −34.9), respectively.

As shown in Table 8, the main differential lipids specific to MRO were 15 TGs, 6 OAHFAs, 4 PGs, and 4 MGDGs. Among the 46 differential lipids, 18 were downregulated and 28 were upregulated. The differential lipids with the highest degrees of upregulation and downregulation in MRO were TG (27:1_18:1_20:3, 36.72) and PE (18:2_18:2, −40.4), respectively.

Table 9 shows that the main differential lipids specific to RO, which include 46 TGs, 18 DGs, and 4 MGDGs. In total, 39 of the 81 differential lipids were downregulated, and 42 were upregulated. The differential lipids with the highest degrees of upregulation and downregulation in RO were DG (27:3e, 28.8) and DG (9:0_12:3, −33.48), respectively.

**Figure 15:**
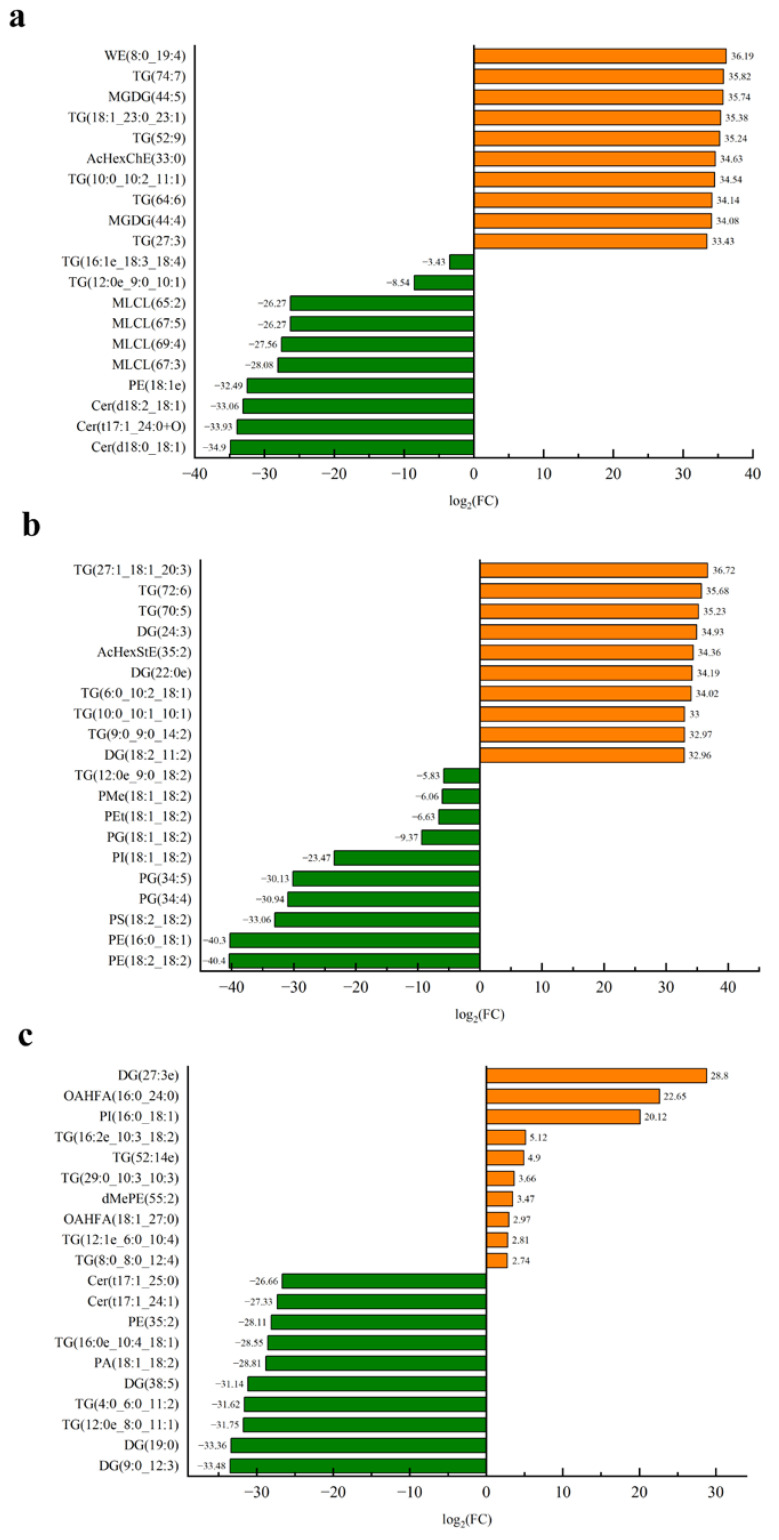
Top 20 lipids of three kinds of camellia oil. (**a**) CO; (**b**) MRO; (**c**) RO.

The differential lipids with the top 10 difference multiples (up and down) were further selected to analyze the difference multiples of the three oils. As shown in Figure 15, the differential lipids were TG, MLCL, and Cer in CO; TG, DG, and PG in MRO; and TG and DG in RO. The upregulation ratio was remarkably greater in CO and MRO than in RO, and the downregulation ratio was greater in MRO and RO than in CO, indicating that the effect of heating on lipid composition in the three kinds of camellia oil was MRO > CO > RO.

#### 3.4.5. Pathways Analyses of Lipids in Camellia Oil

**Figure 16:**
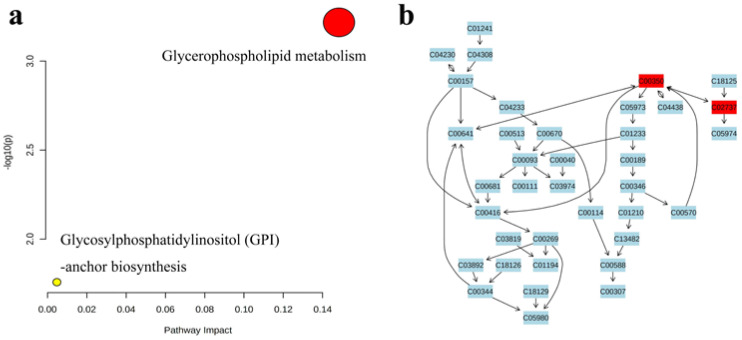
(**a**) Pathway analyses of differential lipids in three camellia oils using three refining methods. (**b**) glycerophospholipid metabolism.

Lipid data with VIP > 1 (667 in total) were imported into MetaboAnalyst 5.0 (https://www.metaboanalyst.ca, accessed on 31 March 2022) for pathway analysis to investigate the effect of the refining method on lipid metabolism after the heating of camellia oil. The results are shown in the form of a bubble chart (Figure 16), with each bubble representing a metabolic pathway [45]. Differential metabolites associated with two metabolic pathways, glycerophospholipid metabolism and glycosylphosphatidylinositol (GPI)-anchor biosynthesis, were observed during heating. Five metabolites were involved in the glycerophospholipid metabolism pathway: PE (34:2), PE (40:0), PE (42:5), PE (44:0), PS (38:4). Four metabolites were involved in the glycosylphosphatidylinositol (GPI)-anchor biosynthesis pathway: PE (34:2), PE (40:0), PE (42:5), and PE (44:0). The abscissa value and bubble size represent the degree of influence in the topology analysis, in which the larger the abscissa value and bubble size, the higher the degree of enrichment. The ordinate value and bubble color represent the *p*-value of the enrichment analysis; the darker the bubble color, the smaller the *p*-value, and the more significant the degree of enrichment [46], which indicated that glycerophospholipid metabolism was the most significant pathway in the pathway analysis results and is important to study in the heating process of oils. Glycerophospholipid metabolism mainly included the transformation between phosphatidylethanolamine (PE, C00350) and phosphatidylserine (PS, C02737), light blue means that those metabolites are not in our data and were used as background for pathway analysis.

PE and PS have important physiological functions. PE, also known as ceruloplasmin, has a variety of physiological functions, such as antioxidant functions, by binding to EPA and DHA [47,48], forming coagulation kinase with proteins that are present in platelets and contributing to blood clotting [49,50,51], maintaining the morphology of the mitochondria [51], and regulating insulin signaling [50]. Originally isolated from a bovine brain by Folch [52] in 1942, PS is widespread and plays an important role in plants and animals. In humans, PS is the main acidic phospholipid in the brain; it can cross the blood–brain barrier and is rapidly absorbed by the body to repair brain damage and improve immunity and memory [53].

**Figure 17:**
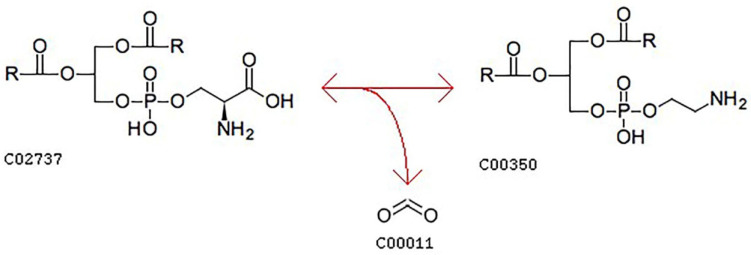
Transformation process between phosphatidylserine (C02737) and phosphatidylethanolamine (C00350) (https://www.genome.jp/entry/R02055, accessed on 31 March 2022). Reprinted with permission from KEGG Database. 2022, Kanehisa Laboratories.

**Figure 18:**
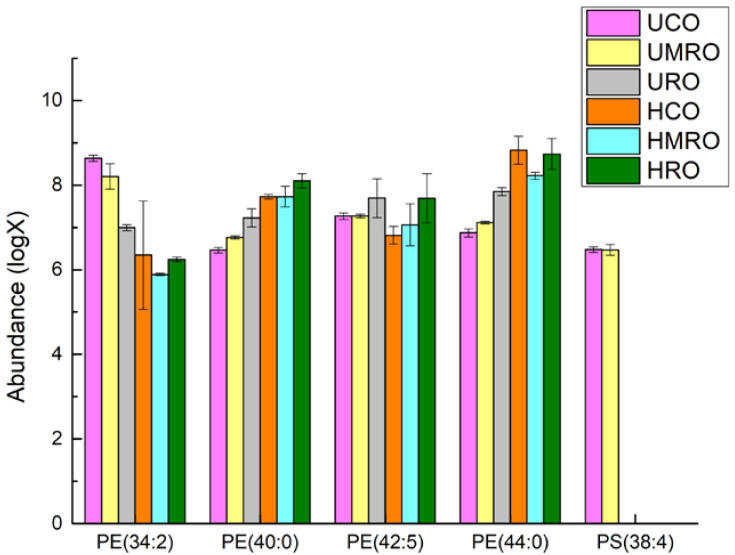
Effects of refining and heating on PE and PS.

A search of the KEGG Database reveals that PS (C02737) is converted to PE (C00350) and CO_2_ via phosphatidylserine decarboxylase (Figure 17) [54]. On the basis of glycerophospholipid metabolism, refining and heating may accelerate the conversion of PS to PE. Figure 18 shows that, compared with UCO, refining reduced the PE (34:2) content but increased the PE (40:0 and 44:0) contents, deep refining increased the PE (42:5) content and reduced the PS (38:4) content, and moderate refining had no effect on these two lipids. Notably, deep refining resulted in the almost complete loss of PS (38:4) content. The effect of heating on lipids was similar to that of refining, that is, heating reduced the PE (34:2) content and increased the PE (40:0 and 44:0) contents. The PE (42:5) content decreased in CO, increased in RO, and remained unchanged in MRO after heating. All three kinds of oils lost almost all of their PS (38:4) when heated. This result showed that moderate refining had little effect on PS content; however, the almost complete loss of PS content after deep refining and heating indicated that these processes had a greater effect on PS than moderate refining. In comparison, moderate refining also resulted in an increase in some PE contents while retaining PS content. In summary, moderate refining is good for retaining the beneficial lipids in camellia oil, and the heating process will remove some beneficial lipids.

## 4. Conclusions

In this study, we used physicochemical and lipidomic analyses combined with a chemometric method to investigate the changes in the oxidation degree and lipid sub-stances of camellia CO, MRO, and RO during heating, and the effects of different refining degrees on camellia oil quality. The deterioration behaviors of three kinds of camellia oil were evaluated by measuring physical parameters, oxidation indexes, and active substance contents. Camellia oil when less refined is less oxidized, probably due to substances such as tocopherols and polyphenols in the camellia oil. However, camellia oil when less refined has a greater change in lipids during the heating process, but moderately refined camellia oil is good for retaining the beneficial lipids in camellia oil. The degree of refining will not only remove impurities from camellia oil, but also eliminate some beneficial components, which may directly affect the oxidation and lipid changes in the subsequent cooking process. Therefore, moderate refining should be advocated in the processing of camellia oil according to the quality of the crude oil and the usage of the oil.

## Figures and Tables

**Table 1:** The initial fatty acid composition of three kinds of camellia oil.

Fatty Acid (%)	CO	MRO	RO
C16:0	7.84 ± 0.11	8.48 ± 0.35	9.41 ± 0.17
C18:0	2.28 ± 0.07	2.38 ± 0.22	2.48 ± 0.18
C18:1	81.79 ± 0.20	79.87 ± 0.75	77.83 ± 0.42
C18:2	7.17 ± 0.05	8.26 ± 0.44	10.28 ± 0.15
C18:3	0.27 ± 0.02	0.45 ± 0.02	ND
C20:1	0.51 ± 0.03	0.53 ± 0.02	ND

ND: not detected.

**Table 2:** Correlation analysis of polyphenols, TOTOX, conjugated alkene, polar components, and tocopherol of camellia oil during heating.

Correlation Coefficient	Tocopherols	Polyphenols	TOTOX	K232	K268	Polar Components
Tocopherols	1					
Polyphenols	0.880 **	1				
TOTOX	−0.737 **	−0.533 **	1			
K232	−0.919 **	−0.727 **	0.877 **	1		
K268	−0.900 **	−0.737 **	0.780 **	0.928 **	1	
Polar components	−0.826 **	−0.652 **	0.838 **	0.955 **	0.813 **	1

** Significantly correlated at the 0.01 level.

**Table 3:** New lipids appeared after the heating process in three kinds of camellia oil.

No.	HCO					HMRO				HRO	
1	AcHexChE (33:0)	21	TG (9:0_9:0_14:2)	41	WE (3:0_20:2)	1	AcHexChE (33:0)	21	TG (6:0_12:2_18:3)	1	DG (27:3e)
2	AcHexCmE (8:0)	22	TG (6:0_10:2_18:1)	42	WE (8:0_19:4)	2	AcHexCmE (8:0)	22	TG (41:1)	2	OAHFA (16:0_24:0)
3	AcHexStE (35:2)	23	TG (14:1e_10:4_10:4)	43	MGDG (35:4)	3	AcHexStE (35:2)	23	TG (18:2e_8:0_18:1)	3	PA (18:1_12:1)
4	AcHexZyE (36:3)	24	TG (6:0_12:2_18:3)	44	MGDG (44:4)	4	AcHexZyE (36:3)	24	TG (19:1_6:0_20:2)		
5	DG (18:2)	25	TG (18:0_9:0_16:0)	45	MGDG (44:5)	5	CmE (0:0)	25	TG (18:1_11:1_18:2)		
6	DG (22:0e)	26	TG (18:2e_8:0_18:1)	46	MGDG (44:6)	6	DG (22:0e)	26	TG (18:1_23:0_23:1)		
7	DG (27:3)	27	TG (52:9)	47	PA (27:1)	7	DG (24:3)	27	TG (28:1_18:1_18:2)		
8	DG (10:0_18:1)	28	TG (57:8)	48	PA (18:1_12:1)	8	DG (18:2_11:2)	28	TG (27:1_18:1_20:3)		
9	DG (18:1_11:2)	29	TG (18:1_23:0_23:1)	49	SQMG (20:1)	9	DG (18:3e_18:2)	29	TG (70:5)		
10	PG (18:1_23:0)	30	TG (28:1_18:1_18:2)	50	dMePE (54:1)	10	PE (62:2)	30	TG (71:3)		
11	TG (27:3)	31	TG (64:6)	51	dMePE (56:2)	11	PG (18:1_23:0)	31	TG (72:6)		
12	TG (6:0_10:2_12:1)	32	TG (27:1_18:1_20:3)			12	TG (4:0_6:0_10:3)	32	TG (30:0_20:2_24:2)		
13	TG (12:0e_6:0_11:3)	33	TG (69:2)			13	TG (29:4)	33	WE (10:0_18:3)		
14	TG (10:0_10:1_10:1)	34	TG (70:2)			14	TG (10:0_10:1_10:1)	34	Cer (d32:4)		
15	TG (6:0_10:2_14:4)	35	TG (70:3)			15	TG (31:5)	35	OAHFA (18:1_19:1)		
16	TG (6:0_11:1_14:2)	36	TG (28:1_18:1_24:2)			16	TG (10:0_10:2_11:4)	36	PA (27:1)		
17	TG (10:0_10:2_11:1)	37	TG (71:3)			17	TG (6:0_6:0_20:2)	37	PA (18:1_12:1)		
18	TG (8:0_11:2_12:1)	38	TG (24:2_23:0_24:2)			18	TG (9:0_9:0_14:2)	38	dMePE (54:1)		
19	TG (10:0_10:2_11:4)	39	TG (72:6)			19	TG (6:0_10:2_18:1)	39	dMePE (56:2)		
20	TG (6:0_6:0_20:2)	40	TG (74:7)			20	TG (14:1e_10:4_10:4)				

**Table 4:** Lipids disappeared after the heating process in three kinds of camellia oil.

No.	HCO	HMRO	HRO
1	Cer (d18:0_18:1)	Cer (d18:2_25:0+O)	BisMePA (22:4_18:2)
2	Cer (d18:2_18:1)	PA (34:1)	DG (19:0)
3	Cer (d18:2_25:0+O)	PE (18:1e)	DG (9:0_12:3)
4	PE (18:1e)	PE (18:0_16:0)	DG (38:5)
5	TG (35:1e)	PE (16:0_18:1)	PA (18:1_18:2)
6	Cer (d34:2+O)	PE (16:0_18:3)	PE (16:0_18:3)
7	Cer (t18:1_18:1)	PE (18:1_18:1)	PE (35:2)
8	Cer (t17:1_24:0+O)	PE (18:1_18:2)	PE (40:5)
9	Cer (t42:1+O)	PE (18:2_18:2)	TG (4:0_6:0_11:2)
10	Cer (t42:2)	Cer (d34:2+O)	TG (12:0e_8:0_11:1)
11	LPE (18:1)	Cer (t18:1_18:1)	TG (35:1e)
12	LPE (18:2)	Cer (t42:1+O)	TG (16:0e_10:4_18:1)
13	MLCL (65:2)	LPE (18:1)	TG (18:1_11:1_18:2)
14	MLCL (67:3)	LPE (18:2)	Cer (t17:1_24:1)
15	MLCL (67:5)	PG (34:4)	Cer (t17:1_25:0)
16	MLCL (69:4)	PG (34:5)	PG (36:5)
17	PG (36:7)	PG (36:7)	
18	PI (18:1_18:2)	PI (18:1_18:2)	
19	PS (38:4)	PS (18:2_18:2)	
20	PS (38:5)	PS (38:4)	
21	PS (38:6)	PS (38:5)	
22		PS (38:6)	

**Table 5:** Statistical table of differential lipids in three kinds of camellia oils after heating.

Types of Lipids	CO			MRO			RO		
	Total Quantity	Upward Adjustment	Downward Adjustment	Total Quantity	Upward Adjustment	Downward Adjustment	Total Quantity	Upward Adjustment	Downward Adjustment
Phospholipids	15	3	12	21	3	18	6	2	4
Sphingolipid	9	1	8	7	2	5	2	0	2
Neutral lipids	29	24	5	29	26	3	72	38	34
Fatty acyl, other phospholipids	11	11	0	16	13	3	3	2	1
Glyceroglycolipid	17	16	1	14	10	4	9	6	3
Derivative lipids	2	2	0	3	3	0	2	2	0
Total	83	57	26	90	57	33	94	50	44

**Table 6:** Statistical table of unique lipids in three kinds of camellia oil after heating.

Types of Lipids	CO			MRO			RO		
	Total Quantity	Upward Adjustment	Downward Adjustment	Total Quantity	Upward Adjustment	Downward Adjustment	Total Quantity	Upward Adjustment	Downward Adjustment
Phospholipids	5	0	5	9	0	9	3	1	2
Sphingolipid	3	0	3	1	1	0	2	0	2
Neutral lipids	19	15	4	21	18	3	68	36	32
Fatty acyl, other phospholipids	4	4	0	9	6	3	3	2	1
Glyceroglycolipid	6	6	0	5	2	3	4	2	2
Derivative lipids	0	0	0	1	1	0	1	1	0
Total	37	25	12	46	28	18	81	42	39

**Table 7:** Statistical table of specific differential lipids in CO.

No.	Compound	*m*/*z*	RT/min	Chemical Formula	VIP	log_2_(FC)
1	AcHexChE (33:0)	1042.9372	20.91	C66H124O7N1	1.1552	34.63
2	AcHexSiE (18:2)	856.7025	18.20	C53H94O7N1	1.1552	−3.32
3	AcHexZyE (36:3)	1076.9216	19.79	C69H122O7N1	1.1550	31.51
4	Cer (d18:0_18:1)	566.5507	14.74	C36H72O3N1	1.1553	−34.90
5	Cer (d18:2_18:1)	562.5194	13.51	C36H68O3N1	1.1553	−33.06
6	Cer (t17:1_24:0+O)	712.6097	16.99	C42H82O7N1	1.1553	−33.93
7	DG (10:0_18:1)	528.4623	12.06	C31H62O5N1	1.1554	32.52
8	DG (18:1_11:2)	538.4466	10.75	C32H60O5N1	1.1553	31.47
9	MGDG (35:4)	763.5366	9.51	C44H75O10	1.1761	27.61
10	MGDG (40:6)	829.5835	16.02	C49H81O10	1.1722	6.86
11	MGDG (42:6)	857.6148	16.53	C51H85O10	1.1692	4.71
12	MGDG (44:4)	889.6774	17.47	C53H93O10	1.1794	34.08
13	MGDG (44:5)	887.6618	16.40	C53H91O10	1.1793	35.74
14	MGDG (47:2)	935.7557	18.48	C56H103O10	1.1635	1.83
15	MLCL (65:2)	1347.9700	13.65	C74H141O16P2	1.1776	−26.27
16	MLCL (67:3)	1373.9857	13.70	C76H143O16P2	1.1788	−28.08
17	MLCL (67:5)	1369.9544	12.57	C76H139O16P2	1.1784	−26.27
18	MLCL (69:4)	1400.0013	13.73	C78H145O16P2	1.1787	−27.56
19	OAHFA (18:1_18:0)	563.5045	17.52	C36H67O4	1.1716	4.27
20	OAHFA (18:1_18:1)	561.4888	16.37	C36H65O4	1.1757	4.97
21	OAHFA (18:2_18:1)	559.4732	15.34	C36H63O4	1.1755	6.86
22	PE (18:1e)	480.3085	3.72	C23H47O7N1P1	1.1553	−32.49
23	TG (10:0_10:2_11:1)	580.4572	12.05	C34H62O6N1	1.1551	34.54
24	TG (12:0e_9:0_10:1)	570.5092	12.29	C34H68O5N1	1.1551	−8.54
25	TG (16:1e_18:3_18:4)	855.6837	17.96	C55H92O5Na1	1.1553	−3.43
26	TG (18:0_9:0_16:0)	754.6919	20.04	C46H92O6N1	1.1552	33.27
27	TG (18:1_23:0_23:1)	1049.9447	20.59	C67H126O6Na1	1.1551	35.38
28	TG (20:2e_14:2_18:3)	857.6993	18.21	C55H94O5Na1	1.1551	−3.20
29	TG (27:3)	529.3500	5.75	C30H50O6Na1	1.1554	33.43
30	TG (28:1_18:1_24:2)	1125.0519	22.77	C73H138O6N1	1.1553	33.22
31	TG (52:9)	845.6654	14.16	C55H89O6	1.1552	35.24
32	TG (6:0_10:2_12:1)	543.3656	7.25	C31H52O6Na1	1.1553	33.14
33	TG (6:0_10:2_14:4)	543.3680	7.25	C33H51O6	1.1554	32.40
34	TG (64:6)	1019.9001	20.93	C67H119O6	1.1552	34.14
35	TG (69:2)	1115.0675	23.21	C72H140O6N1	1.1551	30.80
36	TG (74:7)	1180.0229	21.62	C77H136O6Na1	1.1551	35.82
37	WE (8:0_19:4)	420.3836	9.15	H50C27O2N1	1.1554	36.19

FC: HCO/UCO.

**Table 8:** Statistical table of specific differential lipids in MRO.

No.	Compound	*m*/*z*	RT/Min	Chemical Formula	VIP	log_2_(FC)
1	AcHexStE (35:2)	1075.9263	20.15	C70H123O7	1.1320	34.36
2	Cer (d32:4)	548.4320	7.62	C33H58O5N1	1.1319	25.58
3	CmE (0:0)	401.3778	12.59	C28H49O1	1.1320	32.71
4	DG (18:2_11:2)	536.4310	10.07	C32H58O5N1	1.1319	32.96
5	DG (22:0e)	437.3601	18.55	C25H50O4Na1	1.1319	34.19
6	DG (24:3)	451.3418	16.43	C27H47O5	1.1319	34.93
7	dMePE (41:2)	840.6488	15.41	C48H91O8N1P1	1.2068	9.45
8	MGDG (29:5)	677.4270	7.63	C38H61O10	1.1718	−1.84
9	MGDG (34:2)	753.5522	15.29	C43H77O10	1.1571	−2.21
10	MGDG (41:3)	849.6461	16.84	C50H89O10	1.1988	11.02
11	MGDG (43:6)	871.6305	16.86	C52H87O10	1.1995	10.52
12	OAHFA (18:1_19:1)	575.5045	15.22	C37H67O4	1.2073	31.30
13	OAHFA (18:1_23:0)	633.5827	19.45	C41H77O4	1.1765	6.73
14	OAHFA (18:1_24:0)	647.5984	19.73	C42H79O4	1.1889	4.28
15	OAHFA (18:1_26:1)	673.6140	19.65	C44H81O4	1.1747	6.16
16	OAHFA (18:2_24:0)	645.5827	19.22	C42H77O4	1.1906	6.40
17	OAHFA (38:2)	589.5201	17.19	C38H69O4	1.1838	2.98
18	PA (18:0_18:1)	701.5127	15.04	C39H74O8N0P1	1.1856	−4.44
19	PE (16:0_18:1)	740.5201	12.25	C39H76O8N1P1Na1	1.1319	−40.30
20	PE (18:2_18:2)	740.5225	12.25	C41H75O8N1P1	1.1319	−40.40
21	PEt (18:1_18:2)	725.5127	12.99	C41H74O8N0P1	1.1957	−6.63
22	PG (18:1_18:2)	771.5182	12.13	C42H76O10N0P1	1.1992	−9.37
23	PG (34:4)	741.4712	13.68	C40H70O10N0P1	1.2059	−30.94
24	PG (34:5)	739.4556	12.50	C40H68O10N0P1	1.2078	−30.13
25	PG (40:3)	827.5808	15.02	C46H84O10N0P1	1.1682	−2.41
26	PI (18:1_18:2)	859.5342	11.77	C45H80O13N0P1	1.2086	−23.47
27	PMe (18:1_18:1)	713.5127	13.81	C40H74O8N0P1	1.1958	−4.66
28	PMe (18:1_18:2)	711.4970	12.68	C40H72O8N0P1	1.1750	−6.06
29	PS (18:2_18:2)	782.4978	13.39	C42H73O10N1P1	1.2079	−33.06
30	SQDG (49:0)	1031.7802	19.00	C58H111O12S1	1.1724	−3.94
31	StE (18:3)	673.5918	18.25	C47H77O2	1.1318	−3.12
32	TG (10:0_10:1_10:1)	568.4572	12.19	C33H62O6N1	1.1319	33.00
33	TG (10:0_10:2_11:4)	579.3656	7.50	C34H52O6Na1	1.1318	32.91
34	TG (12:0e_9:0_18:2)	680.6188	16.21	C42H82O5N1	1.1318	−5.83
35	TG (27:1_18:1_20:3)	1052.9580	22.10	C68H126O6N1	1.1319	36.72
36	TG (29:4)	555.3656	6.93	C32H52O6Na1	1.1320	32.36
37	TG (31:5)	576.4259	6.32	C34H58O6N1	1.1320	31.83
38	TG (4:0_6:0_10:3)	426.2850	3.62	C23H40O6N1	1.1319	26.70
39	TG (41:1)	729.6004	18.87	C44H82O6Na1	1.1319	29.71
40	TG (6:0_10:2_18:1)	622.5041	13.90	C37H68O6N1	1.1320	34.02
41	TG (6:0_6:0_24:0)	661.5378	12.66	C39H74O6Na1	1.1319	1.46
42	TG (70:5)	1123.0362	22.64	C73H136O6N1	1.1320	35.23
43	TG (72:6)	1154.0073	21.61	C75H134O6Na1	1.1319	35.68
44	TG (8:0_12:2_18:1)	661.5402	12.65	C41H73O6	1.1319	1.46
45	TG (9:0_11:1_18:1)	680.5824	17.04	C41H78O6N1	1.1319	−2.52
46	TG (9:0_9:0_14:2)	596.4885	13.40	C35H66O6N1	1.1319	32.97

FC: HMRO/UMRO.

**Table 9:** Statistical table of specific differential lipids in RO.

No.	Compound	*m*/*z*	RT/Min	Chemical Formula	VIP	log_2_(FC)
1	AcHexChE (16:0)	804.6712	16.76	C49H90O7N1	1.2766	1.45
2	Cer(t17:1_24:1)	708.6148	16.63	C43H82O6N1	1.2782	−27.33
3	Cer(t17:1_25:0)	724.6461	18.31	C44H86O6N1	1.2795	−26.66
4	DG (14:0_18:2)	587.4646	13.83	C35H64O5Na1	1.2783	−1.94
5	DG (16:0_10:1)	505.3863	7.65	C29H54O5Na1	1.2779	2.15
6	DG (18:1_11:1)	545.4176	8.57	C32H58O5Na1	1.2777	1.26
7	DG (18:1_14:0)	589.4802	14.99	C35H66O5Na1	1.2783	−1.88
8	DG (18:1_18:3)	639.4959	14.37	C39H68O5Na1	1.2756	−1.60
9	DG (18:2_18:2)	639.4959	14.15	C39H68O5Na1	1.2757	−1.55
10	DG (18:3_18:2)	637.4802	13.16	C39H66O5Na1	1.2788	−3.34
11	DG (19:0)	409.2924	18.77	C22H42O5Na1	1.2800	−33.36
12	DG (19:3)	398.2901	4.89	C22H40O5N1	1.2754	2.10
13	DG (20:1_18:2)	664.5875	16.49	C41H78O5N1	1.2755	−1.41
14	DG (22:1_18:2)	692.6188	17.55	C43H82O5N1	1.2755	−1.24
15	DG (27:3e)	496.4360	9.47	C30H58O4N1	1.2763	28.80
16	DG (28:7)	521.3237	6.75	C31H46O5Na1	1.2794	1.91
17	DG (30:5e)	517.4251	6.91	C33H57O4	1.2756	1.85
18	DG (31:8)	561.3550	7.72	C34H50O5Na1	1.2787	1.83
19	DG (38:5)	660.5562	14.22	C41H74O5N1	1.2791	−31.14
20	DG (44:2)	755.6524	16.29	C47H88O5Na1	1.2793	2.23
21	DG (9:0_12:3)	409.2949	18.83	C24H41O5	1.2800	−33.48
22	dMePE (55:2)	1036.8679	19.97	C62H119O8N1P1	1.5310	3.47
23	MGDG (16:0_16:0)	729.5522	14.24	C41H77O10	1.5991	−4.49
24	MGDG (26:2)	641.4270	7.23	C35H61O10	1.4987	−1.13
25	MGDG (31:4)	707.4740	7.19	C40H67O10	1.5067	2.69
26	MGDG (48:6)	941.7087	16.48	C57H97O10	1.4505	2.54
27	OAHFA (16:0_24:0)	621.5827	19.73	C40H77O4	1.6580	22.65
28	OAHFA (18:1_27:0)	689.6453	19.43	C45H85O4	1.5752	2.97
29	PA (18:1_18:2)	716.5225	12.66	C39H75O8N1P1	1.2790	−28.81
30	PE (35:2)	730.5381	12.60	C40H77O8N1P1	1.2796	−28.11
31	PI (16:0_18:1)	835.5342	12.80	C43H80O13N0P1	1.6472	20.12
32	TG (11:0_10:1_10:1)	582.4728	8.15	C34H64O6N1	1.2773	1.29
33	TG (12:0_18:2_18:2)	816.7076	19.32	C51H94O6N1	1.2774	−3.73
34	TG (12:0e_8:0_11:1)	570.5092	12.60	C34H68O5N1	1.2798	−31.75
35	TG (12:1e_6:0_10:4)	503.3731	6.33	C31H51O5	1.2787	2.81
36	TG (12:1e_6:0_11:3)	519.4044	8.15	C32H55O5	1.2761	1.93
37	TG (12:1e_6:0_20:5)	641.5140	15.35	C41H69O5	1.2775	−1.34
38	TG (14:0_18:2_18:2)	844.7389	19.88	C53H98O6N1	1.2796	−1.50
39	TG (14:1e_10:1_10:2)	589.4827	14.95	C37H65O5	1.2783	−1.88
40	TG (16:0_14:2_18:1)	818.7232	19.74	C51H96O6N1	1.2792	−2.10
41	TG (16:0e_10:4_18:1)	727.6235	18.12	C47H83O5	1.2766	−28.55
42	TG (16:2e_10:3_18:2)	740.6188	14.23	C47H82O5N1	1.2791	5.12
43	TG (18:1_10:1_11:2)	695.5221	9.09	C42H72O6Na1	1.2758	1.60
44	TG (18:1_10:3_14:4)	707.5245	8.98	C45H71O6	1.2767	2.29
45	TG (18:1_11:2_12:4)	695.5245	9.08	C44H71O6	1.2762	1.59
46	TG (18:3e_11:1_18:2)	767.6548	16.22	C50H87O5	1.2797	2.09
47	TG (18:3e_12:2_18:2)	779.6548	15.42	C51H87O5	1.2769	1.76
48	TG (18:3e_16:0_18:1)	863.7463	19.69	C55H100O5Na1	1.2757	−1.61
49	TG (18:4_16:0_16:0)	849.6943	16.11	C53H94O6Na1	1.2755	1.45
50	TG (18:4_6:0_18:3)	709.5402	9.45	C45H73O6	1.2768	1.21
51	TG (20:0e_16:0_18:2)	895.8089	20.20	C57H108O5Na1	1.2766	2.48
52	TG (20:2e_10:2_18:1)	805.6680	16.75	C51H90O5Na1	1.2756	1.45
53	TG (20:4e_10:2_20:2)	805.6705	16.76	C53H89O5	1.2756	1.45
54	TG (20:4e_10:4_10:4)	679.4333	7.59	C43H60O5Na1	1.2761	−1.12
55	TG (29:0_10:3_10:3)	809.6654	16.28	C52H89O6	1.2777	3.66
56	TG (31:4)	578.4415	7.55	C34H60O6N1	1.2762	1.06
57	TG (32:1e)	589.4802	15.04	C35H66O5Na1	1.2785	−1.87
58	TG (36:4e)	639.4959	14.27	C39H68O5Na1	1.2765	−1.49
59	TG (36:5e)	637.4802	13.15	C39H66O5Na1	1.2779	−3.26
60	TG (37:5e)	629.5140	15.69	C40H69O5	1.2783	−3.93
61	TG (38:8e)	637.4827	13.13	C41H65O5	1.2779	−3.26
62	TG (4:0_6:0_11:2)	425.2898	18.21	C24H41O6	1.2798	−31.62
63	TG (52:14e)	838.6344	9.96	C55H84O5N1	1.2794	4.90
64	TG (54:8e)	861.7331	19.52	C57H97O5	1.2782	−3.68
65	TG (55:10)	902.7232	19.92	C58H96O6N1	1.2751	−6.08
66	TG (56:8e)	906.7909	18.53	C59H104O5N1	1.2764	−1.19
67	TG (6:0_12:2_12:2)	564.4259	8.98	C33H58O6N1	1.2784	2.14
68	TG (6:0_9:0_18:1)	612.5198	14.69	C36H70O6N1	1.2755	1.02
69	TG (8:0_10:0_11:1)	556.4572	8.66	C32H62O6N1	1.2755	1.04
70	TG (8:0_10:0_11:2)	559.3969	8.14	C32H56O6Na1	1.2770	1.25
71	TG (8:0_11:1_11:2)	566.4415	10.11	C33H60O6N1	1.2759	1.78
72	TG (8:0_11:2_12:3)	559.3993	8.15	C34H55O6	1.2780	1.25
73	TG (8:0_8:0_10:1)	519.3656	7.35	C29H52O6Na1	1.2765	2.74
74	TG (8:0_8:0_12:4)	519.3680	7.35	C31H51O6	1.2765	2.74
75	TG (9:0_10:1_18:1)	666.5667	16.79	C40H76O6N1	1.2754	−1.08
76	TG (9:0_9:0_11:3)	552.4259	6.91	C32H58O6N1	1.2756	2.02
77	TG (9:0_9:0_18:1)	654.5667	17.01	C39H76O6N1	1.2791	−1.47
78	WE (22:5_21:2)	621.5605	17.95	H73C43O2	1.2769	−1.28
79	ZyE (16:1)	621.5605	17.96	C43H73O2	1.2769	−1.28
80	ZyE (21:2)	706.6497	19.65	C48H84O2N1	1.2773	−2.07
81	ZyE (35:6)	894.8062	20.19	C62H104O2N1	1.2752	1.95

FC: HRO/URO.

## Data Availability

Data is contained within the article.

## Abbreviations

CO	crude oil
MRO	moderately refined oil
RO	refined oil
HCO	heated crude oil
HMRO	heated moderately refined oil
HRO	heated refined oil
UCO	unheated crude oil
UMRO	unheated moderately refined oil
URO	unheated refined oil
PV	peroxide Value
*p*-AV	*p*-anisidine value
PCA	principal components analysis
PLS-DA	partial least squares discriminant analysis
OPLS-DA	orthogonal partial least squares discriminant analysis
RF	random forest
SVM	support vector machine
VIP	value importance in projection
FC	fold change
ORA	over representation analysis
SFA	saturated fatty acids
MUFA	monounsaturated fatty acids
PUFA	polyunsaturated fatty acids
UFA	unsaturated fatty acids
TOTOX	total oxidation values
TIC	total Ion Chromatography
TG	triacylglycerol
DG	diacylglycerols
MGDG	monogalactosyl diacylglycerols
PA	phosphatidic acids
Cer	ceramides
MLCL	monolysocardiolipin
PS	phosphatidylserines
LPE	lyso-phosphatidylethanolamines
PE	phosphatidylethanolamines
PG	phosphatidylglycerols
dMePE	dimethylphosphatidylethanolamine
WE	wax esters
SQMG	sulphoquinovosyldiacylglycerols
CmE	campesterol ester
PI	phosphatidylinositols
